# Proximal extension of the deltopectoral approach with ‘bra-strap’ incision: a technical note on a classic technique for acromioclavicular stabilization with step-by-step insights and surgical tips

**DOI:** 10.1016/j.xrrt.2025.06.010

**Published:** 2025-07-05

**Authors:** Erica Lante, Alain Akiki, Geoffroi Lallemand

**Affiliations:** a Department of Orthopedic Surgery and Traumatology, CHUV (Centre Hospitalier Universitaire Vaudois), Lausanne, Switzerland; b Department of Orthopedic Surgery and Traumatology, Riviera Chablais Hospital, Rennaz, Switzerland

**Keywords:** Acromioclavicular dislocation, Acromioclavicular stabilization, Surgical technique, Deltopectoral approach, Deltoid sparing approach, Twin-Tail Tight Rope

Traumatic acromioclavicular (AC) dislocation is one of the most frequent orthopedic conditions (prevalence about 9%), with a higher incidence in young, male patients.^2^,^5^ Surgical options include open,^4^ endoscopic, or even arthroscopically-assisted^7^ procedures, all aiming to stabilize the AC joint but none is considered the gold standard.^3^^,^^4^^,^^6^^,^^8^^,^^9^ Most open techniques share a common approach requiring the crossing or partial detachment of the deltoid to access the deep structures. This technical note proposes the extension of the deltopectoral approach for AC stabilization (Table I). It offers a deltoid-sparing route to the anterior shoulder deep structures. The surgical technique performed at our institution (Riviera Chablais Hospital, Rennaz, Switzerland) for acute AC dislocation (coracoclavicular ligamentoplasty using the Arthrex Twin-Tail Tight Rope system, Arthrex, Naples, FL, USA) will be described. We provide drawings and video from a case of acute AC dislocation (stage IV according to Tossy and Rockwood,^10^^,^^11^ Fig. 1). The patient has given written consent for the scientific dissemination of these materials.

**Table I:** Surgical technique in key points.

Surgical technique in key points
1. Incision: proximal extension of the deltopectoral approach (centered on the coracoid)
2. Development of the deltopectoral interval (medial to the incision)
3. Coracoid localization (central part of the incision)
4. Coracoid tunnel confection and passage of the Tight Rope
5. Localization of the clavicle (proximal part of the incision)
6. Clavicular tunnel confection and passage of the Tight Rope
7. Reducing the acromioclavicular dislocation and tightening the Tight Rope

**Figure 1:**
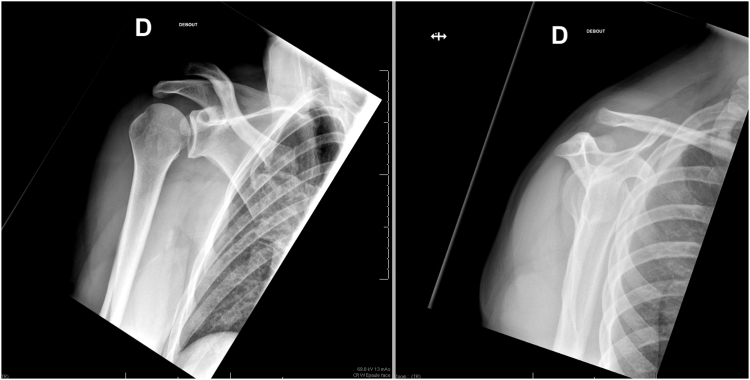
Preoperative right shoulder X-ray (anterior-posterior and Neer views, showing stage IV acromioclavicular dislocation).

## Surgical technique

**Figure 2:**
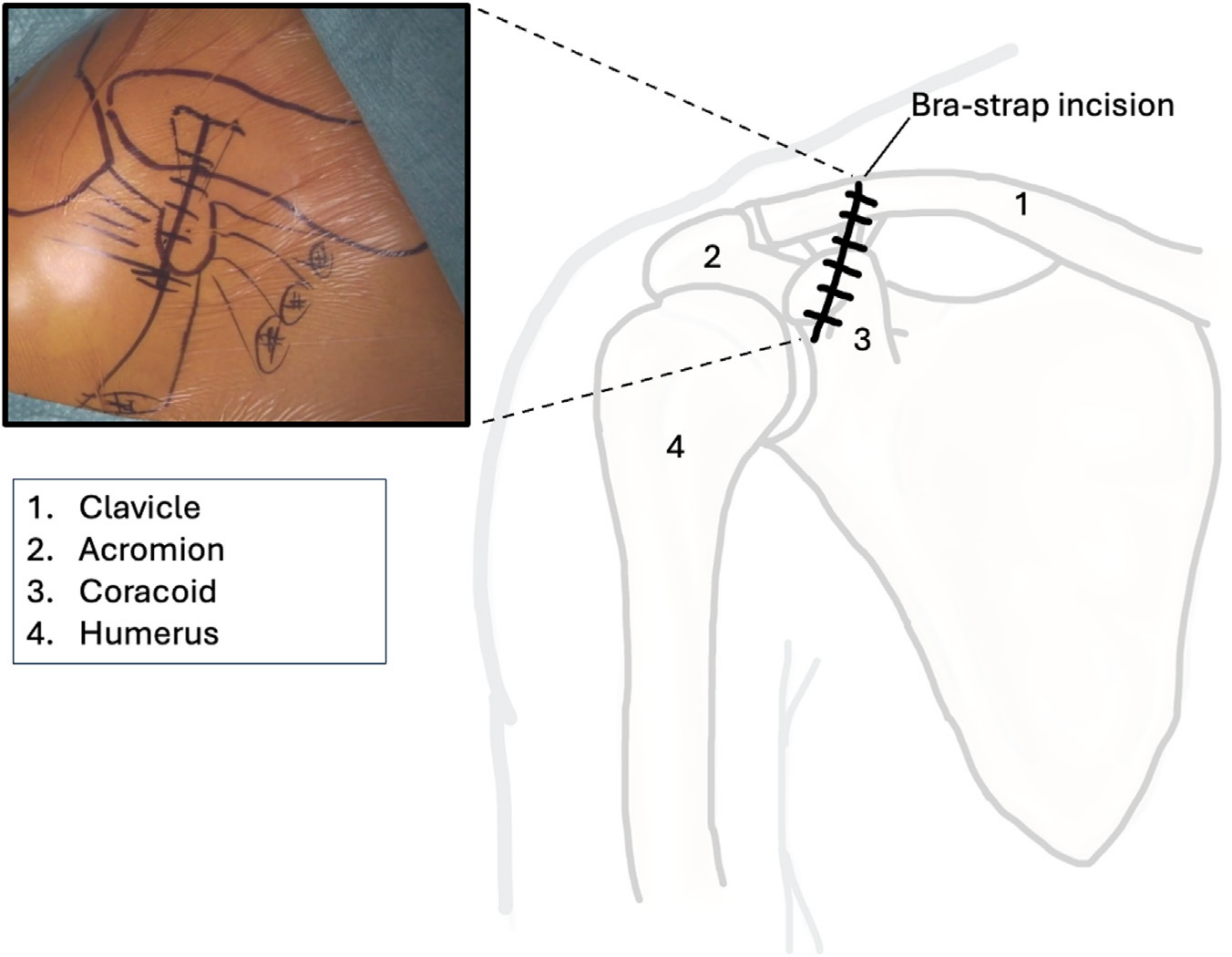
Incision: proximal extension of the deltopectoral approach (centered on the coracoid).

**Figure 3:**
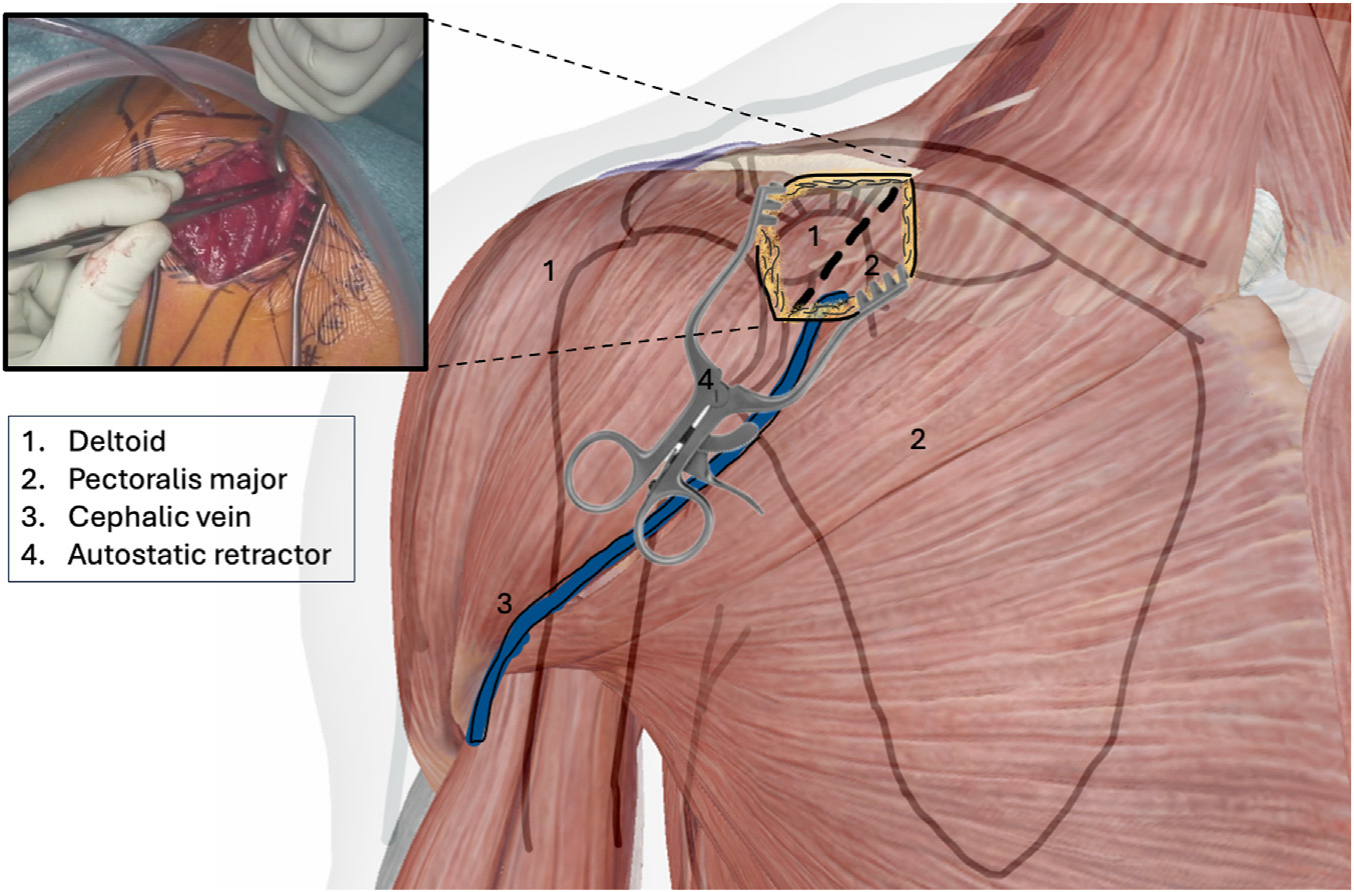
Development of the deltopectoral interval (medial to the incision). This image is a reworked image from Visible Body Suite (version 4.31) [computer software]. (2025). Retrieved January 30, from www.visiblebody.com.

**Figure 4:**
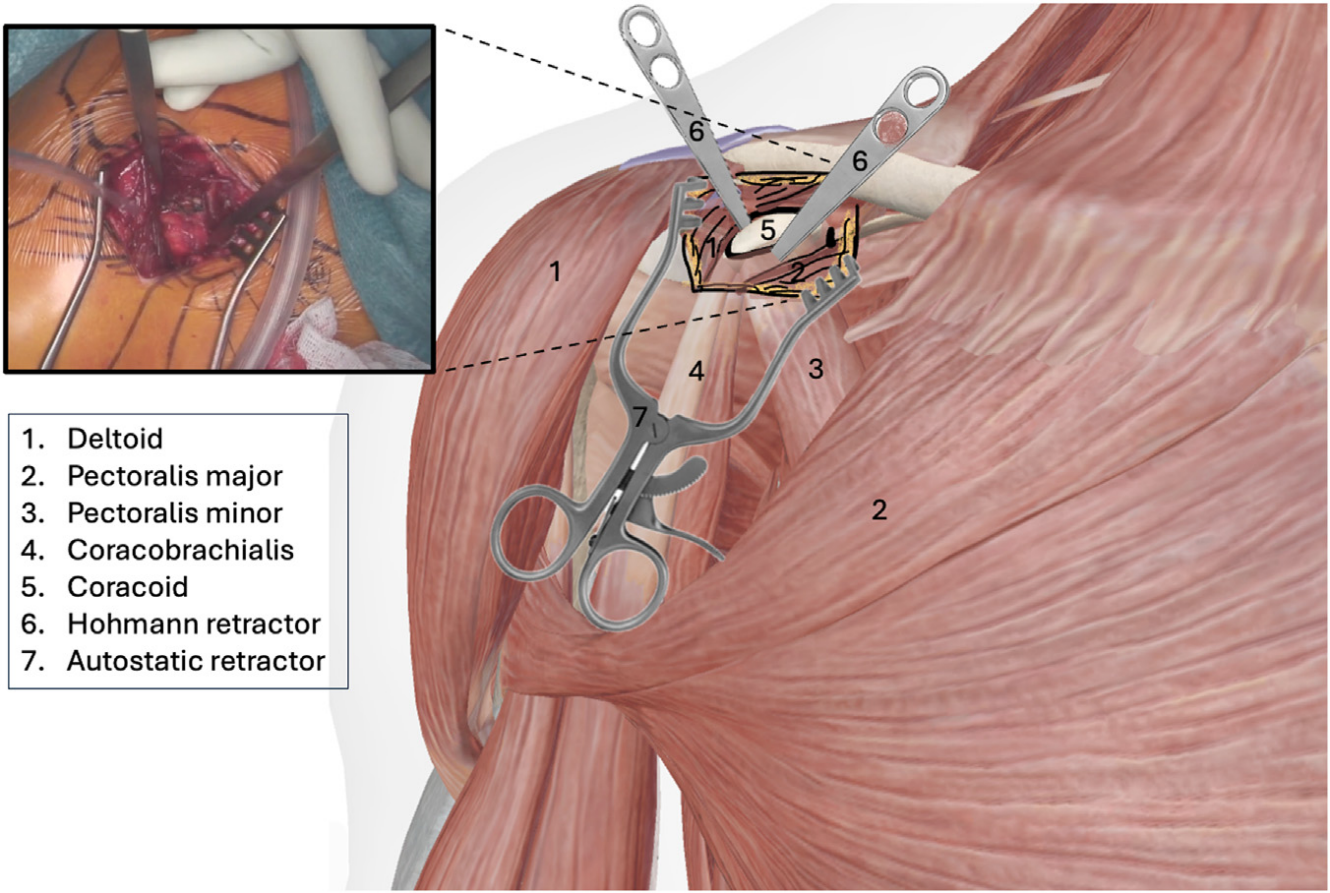
Coracoid localization (central part of the incision). This image is a reworked image from Visible Body Suite (version 4.31) [computer software]. (2025). Retrieved January 30, from www.visiblebody.com.

**Figure 5:**
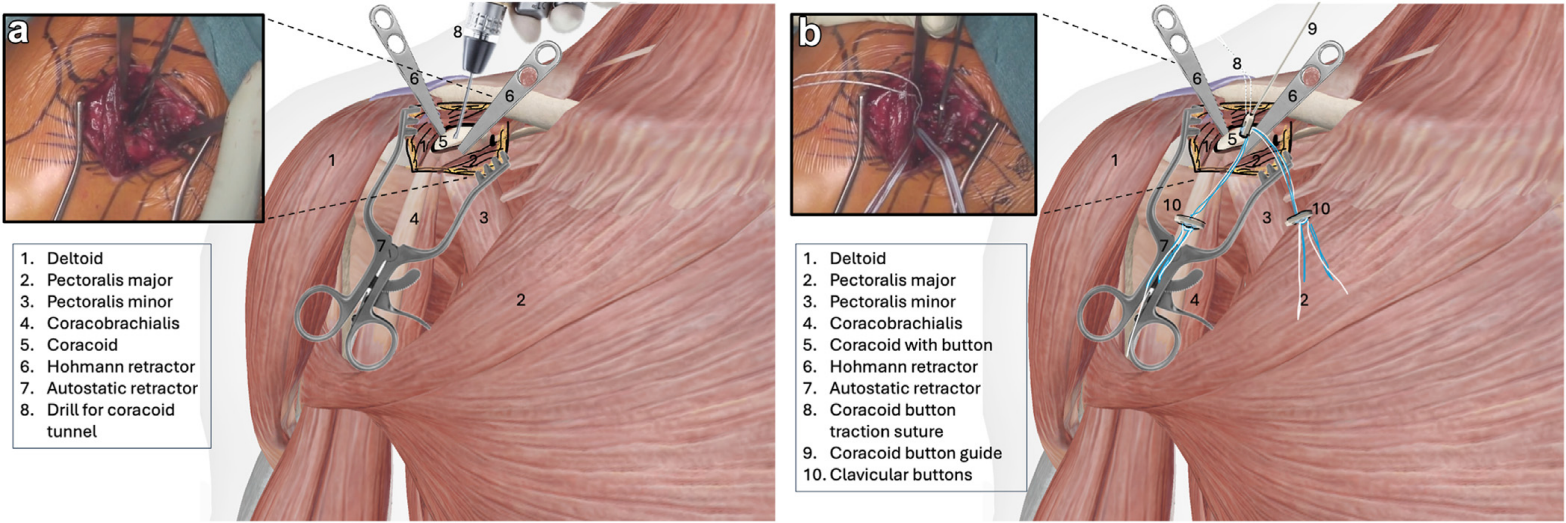
(**a** and **b**). Coracoid tunnel confection (**a**) and passage of the Tight Rope (**b**). This image is a reworked image from Visible Body Suite (version 4.31) [computer software]. (2025). Retrieved January 30, from www.visiblebody.com.

**Figure 6:**
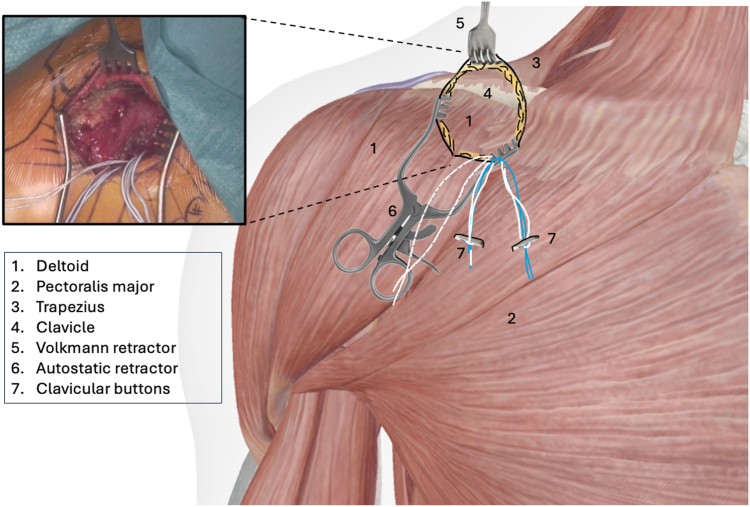
Localization of the clavicle (proximal part of the incision) This image is a reworked image from Visible Body Suite (version 4.31) [computer software]. (2025). Retrieved January 30, from www.visiblebody.com.

**Figure 7:**
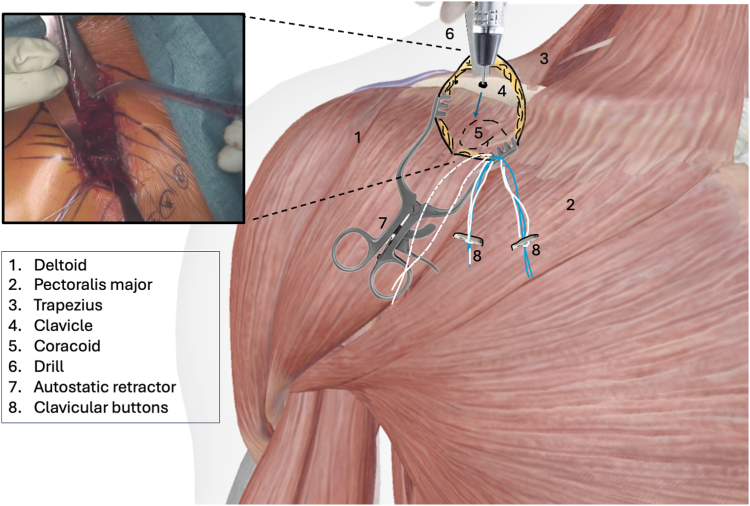
Clavicular tunnel confection and passage of the Tight Rope This image is a reworked image from Visible Body Suite (version 4.31) [computer software]. (2025). Retrieved January 30, from www.visiblebody.com.

**Figure 8:**
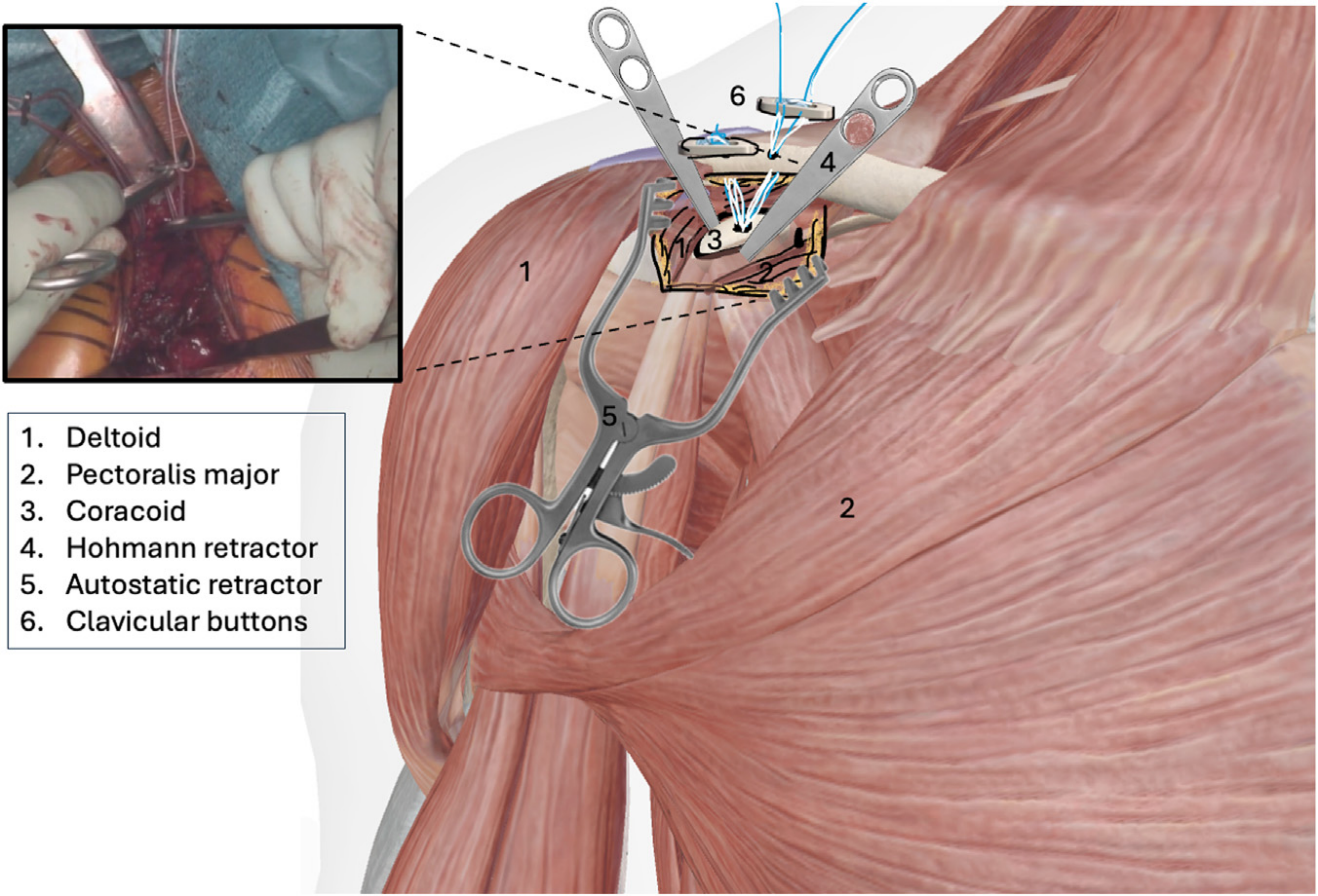
Reduction of the acromioclavicular dislocation and tightening of the Tight Rope This image is a reworked image from Visible Body Suite (version 4.31) [computer software]. (2025). Retrieved January 30, from www.visiblebody.com.

**Figure 9:**
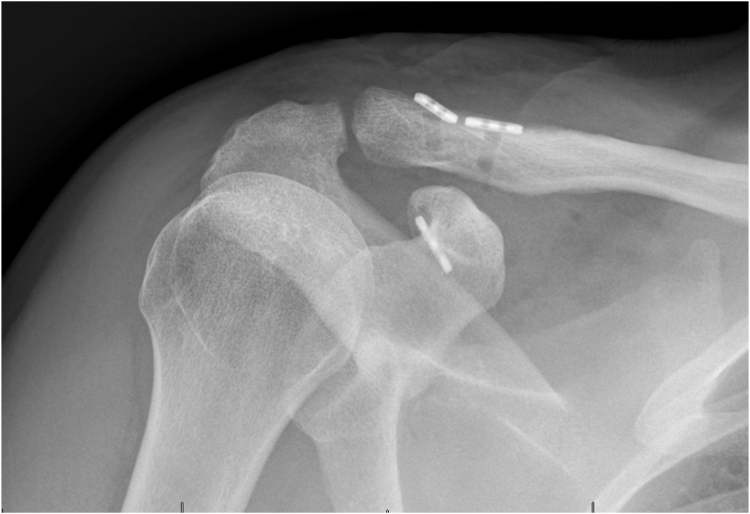
Right shoulder postoperative X ray, Rockwood view.

The procedure is performed under general anesthesia with the patient in beach chair position. The surgeon stands in front of the shoulder, the assistant at the head or side. The patient is placed on a shoulder table without a headboard, allowing the table to bend at the thoracic spine. This increases kyphosis and brings the shoulder forward for optimal AC exposure. The arm lies alongside the body without supports, allowing free movement. The shoulder is extensively washed with Betadine soap, followed by disinfection. The sterile field exposes the distal clavicle, AC joint, coracoid, and shoulder stump. Anatomical landmarks are identified: clavicle, Nevrasier's point, acromion, and coracoid (aligned with the AC joint), along with the AC joint and the coracobrachialis tendon. The coracoclavicular ligaments (trapezoid and conoid) are located at 25.4 and 47.2 mm in males and 22.9 and 42.8 mm in females.^12^ Using a ruler, 2 and 4 cm are measured from the AC joint on the clavicle's upper surface to mark the ligament positions. The incision follows the proximal extension of the deltopectoral groove, centered over the coracoclavicular space, oblique along the groove. It extends from the clavicle's upper edge to 10-20 mm distal to the coracoid. In females, the incision aligns with the bra strap, which is why it is referred to as the “bra-strap incision.” After skin incision (Fig. 2, Video 1), the tissues are disinfected with a Betadine-soaked pad to prevent deep colonization by *Cutibacterium acnes*. A self-retaining retractor is then placed to maintain tension and facilitate subcutaneous tissue dissection. The subcutaneous tissue is dissected down to the clavipectoral fascia using an electric scalpel in coagulation mode, allowing for simultaneous hemostasis. Once the clavipectoral fascia is reached, the deltopectoral interval is dissected using scissors (Fig. 3, Video 1). This sulcus contains adipose tissue and the cephalic vein, which is at risk during the approach. Since the sulcus ends at the coracoid, once identified, dissection shifts proximally toward the clavicle. Next is coracoid release and visualization (Fig. 4, Video 1), achieved by placing 2 small Hohmann retractors medially and laterally. The medial retractor gently retracts the pectoralis major. The lateral 1 retracts the deltoid. The coracoid tip is palpated, and its base is targeted for pin insertion. We proceed with AC stabilization using the Twin-Tail TightRope system from Arthrex (Arthrex, Naples, FL, USA). After placing the pin, a 4.5 mm mesh is used to create the coracoid tunnel (Fig. 5, *a*, Video 1). The Twin-Tail TightRope system includes 1 coracoid button and 2 clavicular buttons. The coracoid button, slightly thicker, is packaged separately. We recommend leaving the clavicular buttons in their packaging to avoid confusion, since they do not anchor properly in the coracoid tunnel. The coracoid button is inserted through the tunnel using its guide and the attached TigerWire (Arthrex, Naples, FL, USA) (Fig. 5, *b*, Video 1). Care must be taken not to touch the TigerWire after insertion, as the button may slip back through the tunnel. Before creating the clavicular tunnels, the clavicular buttons are advanced further along their respective FiberWires (Arthrex, Naples, FL, USA) toward the free end. This provides greater effective length, making them easier to maneuver and tighten during the final steps. Clavicular tunnel preparation follows (Fig. 6, Video 1). The trapezius muscle is partially detached from its clavicular insertion using an electric scalpel and a rasp. Elevating the subcutaneous tissue with a Volkmann's multiretractor can aid this step. The AC joint is identified and serves as the primary landmark for tunnel placement. A needle may be inserted into the joint to maintain visibility throughout the procedure. For optimal reduction, it is essential to release the joint from all adherent tissues. Using a ruler, we measure 2 and 4 cm from the joint and place pins at these distances. These serve as guides for a 4 mm diameter mesh to create convergent clavicular tunnels directed toward the coracoid, which lies anterior to the clavicle and determines the trajectory of both the pins and the mesh (Fig. 7, Video 1). Button passage is performed using the Arthrex Banana-Lasso (Arthrex, Naples, FL, USA). The lasso is inserted into the tunnel from proximal to distal and exits below the clavicle. It is then pulled toward the coracoid using graspers and passed around the white wire connected to 1 clavicular button. The white wire is retrieved through the tunnel to the superior clavicular surface, and the button is guided through its tunnel with clamps, avoiding entrapment in the coracoclavicular ligament stump. The same procedure is repeated for the second clavicular button. It is crucial not to pull the blue wires during this phase, as they are designed for final tightening during AC reduction. Premature traction may lead to button rotation and interfere with correct positioning and passage. AC joint reduction is now performed (Fig. 8, Video 1). At our institution, we use a rasp to low the clavicle, while the contralateral hand elevates the shoulder using the arm as a lever. Once clinical reduction is achieved, the surgeon maintains the position while the assistant progressively tightens the Twin-Tail TightRope wires. Although knots are not strictly required, in our practice we secure the threads to enhance stability. Before knotting or cutting, reduction is verified with intraoperative X-ray. If satisfactory, the TigerWire (coracoid) and the white wires (clavicular) are cut, followed by knotting and cutting of the blue wires. Wound closure begins after stabilization. The deltopectoral interval is not sutured. Closure is performed on the subcutaneous and cutaneous layers using an intradermal absorbable suture. Postoperatively, patients are provided with a brace for analgesic purposes. Active shoulder mobilization is allowed from the first postoperative day, limited to movements below 90 degrees of forward elevation or abduction, and without loading. Load-bearing activities may be resumed three months after surgery. We recommend a follow-up X-rays (Fig. 9) at 1, 3, and 6 months postoperatively to monitor the position of the buttons and rule out loss of reduction or hardware migration.

## Discussion

**Figure 10:**
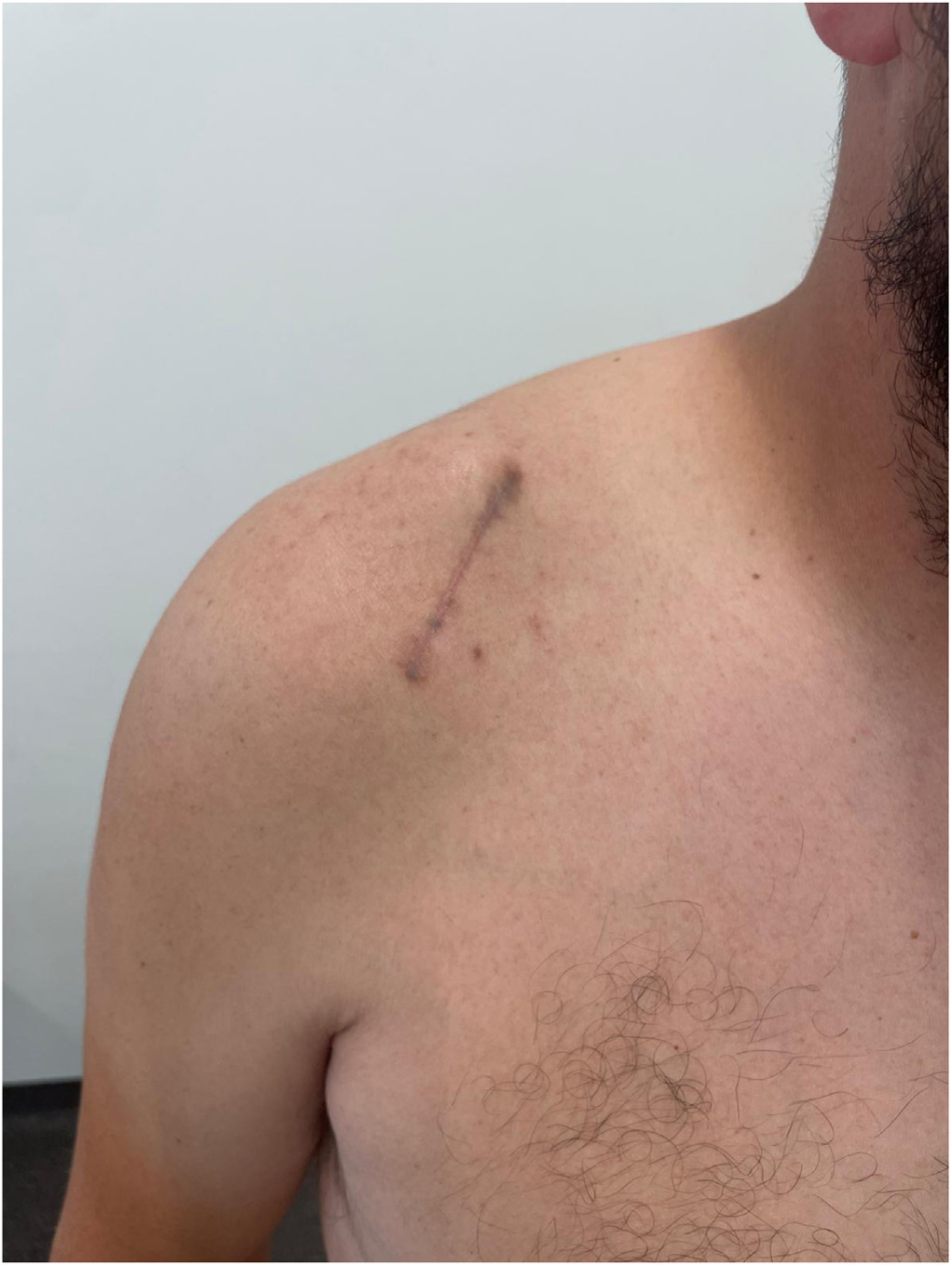
Operative scar 6 weeks postoperative.

This technical note presents the use of the well-known and widely adopted deltopectoral approach for coracoclavicular stabilization. By employing a proximal extension of the deltopectoral approach, the deltoid can be gently retracted laterally, leaving the muscle entirely intact. As a well-established surgical pathway, frequently used in revision shoulder procedures, the deltopectoral approach offers a reliable and familiar route. The detailed procedural description serves as a practical and accessible guide, particularly for surgical trainees. Furthermore, we believe this surgical approach is safe even in the hands of novice surgeons, as it follows natural intermuscular planes. The anatomical landmarks are consistent with those of the classic deltopectoral approach (one of the first taught in surgical training) minimizing the risk of disorientation during the procedure. Surgical exposure is excellent: the coracoid is fully visible, as are the remnants of the conoid and trapezoid ligaments, as well as the AC joint. Direct visualization of both the coracoid and clavicle allows for a lower risk of malreduction and fractures of the clavicle or coracoid, which are often related to misplacement of bone tunnels.^13^ At our institution for acute AC dislocation (within three weeks of injury), we perform a nonanatomical AC stabilization. However, the proposed approach provides excellent exposure for AC reconstruction and grafting procedures, making it highly versatile. While numerous studies compare different stabilization techniques, none to date have directly compared open surgical approaches. In a meta-analysis by Y. Yan et al,^13^ a slight reduction in postoperative pain was reported in favor of the Tight Rope technique compared to the hook clavicular plate. The authors attributed this difference to reduced soft tissue damage with the Tight Rope. In our opinion, the minimal soft tissue disruption afforded by the proximal extension of the deltopectoral approach could contribute to lower postoperative pain. Arthroscopic techniques, while effectives and minimally invasive, include a steep learning curve and significantly higher costs, which limit their accessibility.^1^ Finally, the proximal extension of the deltopectoral approach is also aesthetically favorable (Fig. 10): the scar follows the natural deltopectoral groove, resulting in a discreet and harmonious appearance.

## Conclusion

By combining the reliability of a well-established technique with improved visualization and reduced soft tissue trauma, this muscle-sparing, cost-effective approach offers a safe and efficient solution for AC joint stabilization.

## Acknowledgments

The authors thank Visible Body for kindly providing permission to reproduce anatomical images included in this publication (@visiblebody).

## Disclaimers

Funding: No funding was disclosed by the authors.

Conflicts of interest: The authors, their immediate families, and any research foundation with which they are affiliated have not received any financial payments or other benefits from any commercial entity related to the subject of this article.

## Supplementary data

Video 2

Permission to Use Images Visible Body

## References

[1] Abdelrahman A.A., Ibrahim A., Abdelghaffar K., Ghandour T.M., Eldib D. (2019). Open versus modified arthroscopic treatment of acute acromioclavicular dislocation using a single tight rope: randomized comparative study of clinical outcome and cost-effectiveness. J Shoulder Elbow Surg.

[2] Allman F.L. (1967). Fractures and ligamentous injuries of the clavicle and its articulation. J Bone Joint Surg Am.

[3] Berthold D.P., Muench L.N., Dyrna F., Mazzocca A.D., Garvin P., Voss A. (2022). Current concepts in acromioclavicular joint (AC) instability – a proposed treatment algorithm for acute and chronic AC-joint surgery. BMC Musculoskelet Disord.

[4] Bostrӧm Windhamre H., von Heideken J., Une-Larsson V., Ekstrӧm W., Ekelund A. (2022). No difference in clinical outcome at 2-year follow-up in patients with type III and V acromioclavicular joint dislocation treated with hook plate or physiotherapy: a randomized controlled trial. J Shoulder Elbow Surg.

[5] Chillemi C., Franceschini V., Dei Giudici L., Alibardi A., Salate Santone F., Ramos Alday L.J. (2013). Epidemiology of isolated acromioclavicular joint dislocation. Emerg Med Int.

[6] Dyrna F., Imhoff F.B., Haller B., Braun S., Obopilwe E., Apostolakos J.M. (2018). Primary stability of an acromioclavicular joint repair is affected by the type of additional reconstruction of the acromioclavicular capsule. Am J Sports Med.

[7] Hashiguchi H., Iwashita S., Abe K., Sonoki K., Yoneda M., Takai S. (2018). Arthroscopic coracoclavicular ligament reconstruction for acromioclavicular joint dislocation [internet]. http://www2.nms.ac.jp/jnms/.

[8] Lindborg C.M., Smith R.D., Reihl A.M., Bacevich B.M., Cote M., O’Donnell E. (2024). Current concepts in management of acromioclavicular joint injury. J Clin Med.

[9] North A.S., Wilkinson T. (2018). Surgical reconstruction of the acromioclavicular joint: can we identify the optimal approach?. Strategies Trauma Limb Reconstr.

[10] Rockwood CA WGYD (1998).

[11] Tossy J.D., Mead N.C., Sigmond H.M. (1963). Acromioclavicular separations: useful and practical classification for treatment. Clin Orthop Relat Res.

[12] Xin L., Luo J., Chen M., He B., Tang B., Tang C. (2021). Anatomy and correlation of the coracoid process and coracoclavicular ligament based on three-dimensional computed tomography reconstruction and magnetic resonance imaging. Med Sci Monit.

[13] Yan Y., Liao M., Lai H., Xu Z., Chen H., Huang W. (2023). Comparison of effectiveness and safety in treating acute acromioclavicular joint dislocation with five different surgical procedures: a systematic review and network meta-analysis. Orthop Surg.

