# Supplementary material for: Proximal extension of the deltopectoral approach with ‘bra-strap’ incision: a technical note on a classic technique for acromioclavicular stabilization with step-by-step insights and surgical tips
Source: JSES Rev Rep Tech. 2025 Jul 5;5(4):1147–53. doi: 10.1016/j.xrrt.2025.06.010 (PMC12573486; doi:10.1016/j.xrrt.2025.06.010)
Supplement: Permission to Use Images Visible Body [file mmc3.pdf]

Permission to use content from Visible Body products

The team at Visible Body is delighted by all the interest in using still images and video clips taken from our products and used by our customers to create additional learning materials. The policies listed here are created to strike a balance between protecting our intellectual property and supporting the goals we share with customers of furthering learning. This policy does not change any of the terms of the Visible Body User Agreement or your service plan.

Institutional plan holders and Courseware students

Instructors, students, and healthcare professionals who have an active Visible Body Courseware account or are at an institution with a site or seat subscription to one of our products, can use still images and video clips within their academic communities with wide latitude, as described below.

Permission to use Visible Body products in educational materials (like course handouts, a paper or presentation) is granted at no cost, provided that:

- The course requires Visible Body Courseware with at least 15 students subscribed or the institution has an institutional subscription to one of our products
- Materials are circulated exclusively amongst students and instructors with access to active accounts
- Materials are not sold in any form
- Content includes "Courtesy of Visible Body" displayed with the image or video.
- Materials do not support ad revenue

Note: Restricted instructor support materials, including answers to quiz and lab manual questions, are available to instructors who, subject to approval:

- Are teaching faculty at an accredited institution with an institutional subscription to Courseware or VB Suite
- Are teaching faculty at an accredited institution that requires Visible Body Courseware with at least 15 students subscribed.

These materials are excluded always from permissions grants and are not to be shared.

Classroom/Professional subscription plan holders

Classroom/ professional subscription plan holders have the same sharing permissions as the general public.

Not available under this plan:

- Restricted instructor support materials, including answers to quiz and lab manual questions
- Option to use VB images or links in handouts/student materials

General public for educational non-commercial purposes

On a limited basis and with restrictions, we do grant no-cost permissions to share our intellectual property in a public setting to be viewed by a general audience. This includes content from any of our for-sale products as well as our free content available on the web. Please read these limitations and restrictions carefully.

Permission is granted to use up to 5 images / 20 seconds of video per entity per year with these restrictions:

- Images or video are not sold in any form
- Images or video are used to explain anatomy, physiology, or pathology--not to explain a for-sale product
- With the exception of academic journals, the print, website, or social media in which our intellectual property is used is not sold, ad supported, nor does it have access restricted by a pay wall.
- Credit is always shown:
    - If print or video, use "Image courtesy of Visible Body" displayed with the image or video.
    - If a website or social media either (1) Include "Image courtesy of Visible Body" and hyperlink Visible Body to www.visiblebody.com (2) Or include "Thanks to @visiblebody" or "Thanks to Visible Body" with @visiblebody or Visible Body hyperlinked.
    - For academic journals the citation can be as follows:
      [product name] (release number) [Computer Software]. (release year). [date of retrieval and company url.]
      Here is an example: Visible Body Suite (Version 4.31) [Computer software]. (2023). Retrieved January 30, 2023 from www.visiblebody.com

Note: These permissions are granted at the sole discretion of Visible Body and may be revoked at any time. We recommend you check back here from time to time. Our team has worked to make these explanations as clear as possible. If you have any questions about these guidelines, please seek independent legal advice. Our support team is not able to provide advice on specific use cases.

Licensing images or videos

We do not currently have a program for licensing images or videos for use outside the scenarios and limits outlined above. Please do not contact us about a fee for licensing images or videos from our products; these requests are currently declined.

Commercial distribution and partnerships

We do have a number of partnership and distribution deals. These agreements are annual recurring contracts that include:

- Distributing current versions of our products
- Distributing white label versions of our products
- Distributing adapted versions of our products

The entry point for commercial distribution and partnerships is currently $7,500 a year. Contact support at support@visiblebody.com to be directed to a sales person.
